# Survey of Nonprescription Medication and Antibiotic Use in Patients with Stevens-Johnson Syndrome, Toxic Epidermal Necrolysis, and Overlap Syndrome

**DOI:** 10.3390/antibiotics7010011

**Published:** 2018-02-01

**Authors:** Katherine J. Sullivan, Meghan N. Jeffres, Robert P. Dellavalle, Robert Valuck, Heather D. Anderson

**Affiliations:** 1 Department of Pharmaceutical Sciences, University of Colorado Skaggs School of Pharmacy and Pharmaceutical Sciences, University of Colorado Anschutz Medical Campus, Aurora, CO 80045, USA; 2 Department of Clinical Pharmacy, University of Colorado Skaggs School of Pharmacy and Pharmaceutical Sciences, University of Colorado Anschutz Medical Campus, Aurora, CO 80045, USA; Meghan.Jeffres@UCDenver.edu (M.N.J.); Robert.Valuck@UCDenver.edu (R.V.); Heather.Anderson@UCDenver.edu (H.D.A.); 3 Dermatology Section, US Department of Veterans Affairs Eastern Colorado Health Care System, Denver, CO 80220, USA; Robert.dellavalle@va.gov; 4 Department of Dermatology, University of Colorado School of Medicine, University of Colorado Anschutz Medical Campus, Aurora, CO 80045, USA

**Keywords:** Stevens-Johnson syndrome, toxic epidermal necrolysis, nonprescription medications, over the counter medications, antibiotics

## Abstract

Stevens-Johnson syndrome (SJS), toxic epidermal necrolysis (TEN), and overlap syndrome (SJS-TEN) are rare, serious skin and mucosa break-down conditions frequently associated with antibiotic use. The role of nonprescription medications alone, or in combination with antibiotics in triggering SJS/TEN, is largely unknown. This study summarized data collected from patient surveys about nonprescription and antibiotic use prior to a SJS/TEN diagnosis. The survey was administered online to members of the U.S. SJS Foundation who had been diagnosed with SJS/TEN or were the parent of a child who had been diagnosed with SJS/TEN. Respondents were asked about nonprescription medications taken within the year before diagnosis, and the approximate point in time before diagnosis that they had taken them. They were also asked about specific prescription medications, including antibiotics, that they took before diagnosis. An estimated 4500 patients received an invitation to complete the survey. 251 patients completed it, resulting in a response rate of 5.6%. The mean age of respondents was 43 years (SD (standard deviation) = 17.3) and 70% were female. 32.3% of respondents indicated that a prescription antibiotic triggered their reaction. 14.1% indicated a nonprescription medication had triggered their SJS/TEN, and 18.1% said a nonprescription medication may have triggered their SJS/TEN. 85.5% of respondents said they took a nonprescription medication within three months of their SJS/TEN diagnosis. Of those respondents who reported that an antibiotic triggered their SJS/TEN, 35.2% reported taking a nonprescription medication within the three months prior to their diagnosis. This survey captured valuable information about nonprescription and antibiotic use in SJS/TEN patients. It is important for future studies to estimate the impact of antibiotics on SJS/TEN, and account for nonprescription medication use in that relationship.

## 1. Introduction

Stevens-Johnson syndrome (SJS), overlap syndrome (SJS-TEN), and toxic epidermal necrolysis (TEN) are rare disorders of the skin and mucosa that are most often the result of an adverse medication reaction [1,2,3]. These disorders lie on a spectrum of severity that is characterized by the amount of skin sloughing that occurs over the body; SJS is the least severe, with less than 10% skin sloughing, and TEN is the most severe, with greater than 30% skin sloughing [2]. While these disorders are rare, with an incidence of one to seven cases per million per year in the United States, they have high mortality rates. Mortality ranges from 10% for SJS to 50% for TEN [1,2,4,5].

Several prescription medications have been associated with SJS/TEN, including several types of antibiotics. These include macrolides, quinolones, cephalosporins, tetracyclines, aminopenicillins, and sulfonamides [2,3]. The association of prescription medications with SJS/TEN has been examined in several European countries using case-control studies [6,7]. In the United States, a disproportionality analysis of the U.S. Food and Drug Administration (FDA) Adverse Event Reporting System (AERS) database was undertaken to identify a comprehensive list of medications that were suspected to be associated with SJS/TEN [3].

Though the association of prescription medications with SJS/TEN has been examined in several studies, the association of nonprescription medications with SJS/TEN is not well studied in the U.S. It is estimated that approximately 79% of the U.S. general population takes a nonprescription medication in a given year [8]. This high prevalence of nonprescription medication use in the U.S. suggests that nonprescription medications could play a role in triggering SJS/TEN. Due to the nature of some illnesses for which antibiotics are prescribed, it is possible that many individuals might take nonprescription medications during illnesses requiring antibiotics. Nonprescription medications could be a primary risk factor for SJS/TEN, or they could be part of a drug-drug interaction for patients taking more than one medication, particularly since polypharmacy rates seem to be increasing in the U.S. [9].

The impact of nonprescription medications on SJS/TEN is not well documented in the U.S. SJS/TEN population. In order to better understand the impact of nonprescription medications on SJS/TEN, we developed a survey to capture nonprescription medication use in the U.S. SJS/TEN population. Prescription medications, including antibiotics, were also captured in this survey. The aim of this study was to summarize data collected from the survey about nonprescription medication and antibiotic use prior to a SJS/TEN diagnosis.

## 2. Materials and Methods

In November 2016, the SJS Foundation administered a survey to their registry of SJS, SJS-TEN, and TEN patients. Patients received an initial email in November 2016 containing the link to the online survey, and then received a reminder email in December 2016. The survey closed in January 2017. This survey was developed in Qualtrics, a password-protected online survey software tool [10]. All responses to the survey were confidential and anonymous. The study was approved by the Colorado Multiple Institutional Review Board (protocol 16-2309).

This survey was developed by a committee that included a pharmacist, a dermatologist, and three pharmaceutical outcomes researchers at the University of Colorado, Anschutz Medical Campus. The survey was pilot tested in September 2016 by attendants of the joint meeting of the 7th International Dermato-Epidemiology Association (IDEA) Congress and Keratinocyte Carcinoma Consortium (KeraCon) Meeting. After incorporating feedback from the pilot test, the survey was sent to eligible respondents.

Eligible respondents included individuals who were diagnosed with SJS/TEN or are the parent of a child who was diagnosed with SJS/TEN. Respondents were asked about specific nonprescription medications taken within a year before diagnosis, including pain relievers, cold and flu medications, antacids, laxatives, vitamins, and supplements, and the approximate point in time before diagnosis that they had taken them. They were also asked about the specific prescription medications, including antibiotics, taken before diagnosis. Respondents were asked if their SJS/TEN was triggered by a certain nonprescription or prescription medication, and if so which one(s). Table 1 shows a high-level overview of the questions in the survey. Data from the survey were summarized using descriptive statistics, and a chi-square test was used to test for an association between antibiotics triggering SJS/TEN and taking nonprescription medications. All statistics were calculated using SAS, Version 9.4 [11].

## 3. Results

In November 2016, an estimated 4500 SJS/TEN patients, all members of the U.S. SJS Foundation, received an email invitation to complete the survey. 251 patients completed the survey, resulting in a response rate of 5.6%. The survey closed in January 2017. The average age of respondents was 43 years (SD = 17.3). A majority of respondents were female (70%) and white (85%). Table 2 shows a more detailed list of respondent demographics. Note that while 251 patients responded to the survey, not all respondents answered all survey questions.

**Figure 1:**
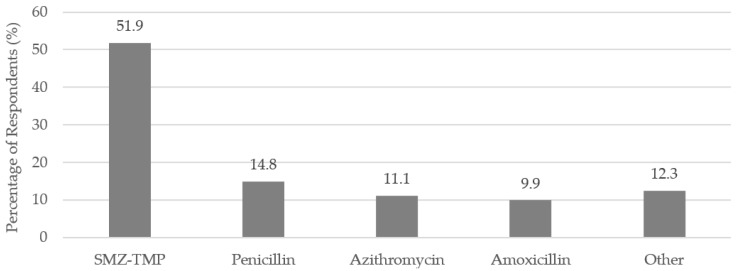
Antibiotics most often reported by respondents as the trigger of their SJS/TEN (*n* = 81); SMZ-TMP = sulfamethoxazole-trimethoprim.

Almost one-third (32.3%) of the respondents indicated that a prescription antibiotic triggered their SJS/TEN (*n* = 81). The most commonly reported antibiotics were sulfamethoxazole-trimethoprim (SMZ-TMP), penicillin, azithromycin, and amoxicillin (Figure 1).

**Figure 2:**
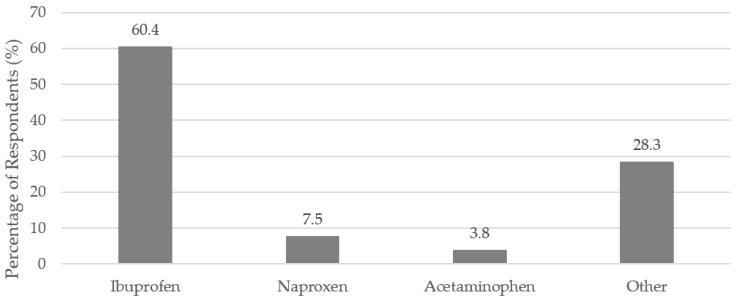
Nonprescription medications reported by respondents as possible triggers of their SJS/TEN (*n* = 53).

Eighty-five percent (*n* = 141 out of 165 respondents) reported taking a nonprescription medication within the three months leading up to their SJS/TEN. Ibuprofen, naproxen, and acetaminophen were reported as possible triggers of SJS/TEN. Figure 2 shows the percentage of respondents reporting these specific nonprescription medications. When asked if a nonprescription medication triggered their SJS/TEN, about 14.1% (*n* = 25) of respondents reported that a nonprescription medication triggered their SJS/TEN, and about 18.1% (*n* = 32) reported that a nonprescription medication maybe triggered their SJS/TEN (177 respondents answered the question).

Of those respondents who reported that an antibiotic triggered their SJS/TEN (*n* = 81), 35.2% reported taking a nonprescription medication within the three months prior to their diagnosis. A Pearson chi-square test for independence indicated that there was not a statistically significant association between antibiotics triggering SJS/TEN, and taking nonprescription medications within the three months before SJS/TEN (*p* = 0.27). 

## 4. Discussion

While the role of prescription medications in triggering SJS/TEN has been studied to an extent in the U.S., the role of nonprescription medications alone or in combination with antibiotics has not been studied. This survey acted as a first step in collecting information about nonprescription and prescription medication use in a U.S. SJS/TEN population, so that these associations might be better understood in the future.

Among other prescription medications, antibiotics have been found to be associated with SJS/TEN in previous studies [3,6]. Specifically, sulfamethoxazole-trimethoprim (SMZ-TMP) was an antibiotic found to be “highly suspect” in triggering SJS/TEN in one of the largest SJS/TEN studies in the U.S. [3]. The survey data reflected this finding. Over 50% of respondents cited SMZ-TMP as a trigger of their SJS/TEN in the survey. 

Nonprescription pain medications are the most common nonprescription medications used in the U.S. Acetaminophen is the most commonly taken nonprescription medication, followed by ibuprofen [9]. Both were reported as possible triggers of SJS/TEN by the patients surveyed. Interestingly, ibuprofen was the most commonly reported nonprescription medication that might have been a trigger of SJS/TEN, while acetaminophen was the third-most commonly reported. This is different from reported use of nonprescription pain medications in the general population [9]. Further studies could be done to better understand if ibuprofen, and other nonprescription medications, are associated with SJS/TEN. There are published case reports that cite ibuprofen, naproxen, and acetaminophen as suspected causative medications for SJS/TEN [12,13,14]. One of the largest case-control studies that looked at medications associated with SJS/TEN, by Mockenhaupt et al., found no significant risk between propionic acid derivatives (such as ibuprofen and naproxen) with SJS/TEN in Europe, but did find a significant association with acetaminophen [6]. The associations between these nonprescription medications and SJS/TEN are still unclear in the U.S.

Retrospective surveys, such as this one, have inherent biases. Recall bias can certainly have an impact on survey results. Respondents’ recollection of which medication triggered their reaction is likely to have been accurate in our survey, especially because they need to remember to avoid that medication in the future so that the same reaction does not occur. Remembering other medications that did not necessarily trigger their SJS/TEN, but that they took prior to their SJS/TEN diagnosis, might have been more difficult to remember; however, it is likely that the respondents discussed with their healthcare providers all medications they had taken prior to their reaction. To reduce recall bias, the respondents were given the option in our survey to report that they simply did not remember which medications they took so that they did not select a medication just for the sake of answering the question, even if they did not necessarily remember taking it. If respondents did select a medication for the sake of answering the question, then this type of recall bias could potentially result in an overestimation of the number of respondents who took any given medication. We do not believe this to be the case for our survey. It is possible that we underestimated the number of respondents who took any given medication due to respondents not remembering if they took that medication during the specified timeframe prior to their SJS/TEN.

Despite the low response rate and potential biases discussed above, it is thought that this survey sample is a close approximation of the general SJS/TEN population. We compared previously published data to the gender and age distributions of our survey respondents. Our survey did have a slightly higher proportion of women than what has been measured in other studies on SJS/TEN (70%). Previously published data varies, but many studies show that the proportion of females with SJS/TEN is higher than males. For example, Mockenhaupt et al. found that 62% of their cases were female [6]. A more recent study, by Hsu et al., looked at a representative sample of all hospitalizations in the U.S. and found that 58.7% of cases were female [15]. The average age of our survey respondents was approximately 43 years. The average age of SJS/TEN cases also varies slightly across different studies. For example, Hsu et al. note that the average ages for SJS, overlap syndrome, and TEN were 57.6, 55.8, and 59.6, respectively [15]. Frey et al. cite the mean age of their SJS/TEN sample from a primary care database in the UK as 37.5 years [16]. In their case-control study, Mockenhaupt et al. cite the median age of their SJS/TEN sample as 50 years [6]. It is, therefore, not believed that our survey respondents are different from the general SJS/TEN population.

## 5. Conclusions

The impact of nonprescription medications on the relationship between antibiotics and SJS/TEN is still unclear. A simple chi-square test for independence indicated that there was not an association between taking nonprescription medications and reporting antibiotics as a trigger of SJS/TEN. Though no association was found in this limited sample, further studies are needed. Even with a small sample size and low response rate, the information that was captured in the survey is still very valuable; it is especially important because very little is known about nonprescription medication use in SJS/TEN patients in the U.S.

This survey has provided information about nonprescription and prescription medication use in SJS/TEN patients. Though the information from this survey is an important first step in understanding the role of nonprescription medications and antibiotics in SJS/TEN, a causative relationship between antibiotics and nonprescription medications should not be interpreted from the results of this survey. A cross-sectional survey like this cannot address causative associations between antibiotics and nonprescription medications and SJS/TEN. Rather, it can generate important questions to investigate further. Further research needs to be done to examine any correlations that might exist. In the future, an associative study, such as a case-control study, would be a logical next step in understanding the impact of nonprescription medications on the relationship between SJS/TEN and prescription antibiotic use in the U.S. It is important for future studies to estimate the impact of antibiotics on SJS/TEN, and account for nonprescription medication use in that relationship.

## Figures and Tables

**Table 1:** High-level overview of questions asked in the survey *.

Were you or someone you know diagnosed with Stevens-Johnson syndrome (SJS), overlap syndrome (SJS-TEN), or toxic epidermal necrolysis (TEN)? When? Did you take any nonprescription (i.e., over-the-counter) medications the year before being diagnosed with SJS or TEN? Specifically, when (3, 4–6, 7–9, 10–12 months prior to diagnosis)? To the best of your ability, please check all of the time periods that you remember taking the following nonprescription medications (brand names of the following types of medications were listed: pain, allergy, cold/flu, laxative, antacid medications, vitamins, and supplements). Time periods include: time of diagnosis, within 3, 4–6, 7–9, 10–12 months before diagnosis, do not remember when I took it, do not remember if I took it, and did not take the medication. Did a nonprescription (i.e., over-the-counter) medication trigger your SJS or TEN? If so, which one(s)? Did a prescription medication trigger your SJS or TEN? If so, which one(s)? Other than the prescription medication(s) that may have triggered your SJS or TEN, were you taking any other prescription medications when you were diagnosed? If so, which one(s)? Did you have any of the following illnesses when you were diagnosed with SJS or TEN? (Options included: herpes, pneumonia, HIV, AIDS, hepatitis, erythema multiforme, mycoplasma pneumonia, other, none of the above) Have you been diagnosed with SJS or TEN more than once? If so, how many times? Demographics-related questions including: age, gender, type of health insurance, state, race, ethnicity, education level How many alcoholic drinks did you consume the week prior to your diagnosis of SJS or TEN? Around the time that you were diagnosed, did you use any tobacco products? Around the time that you were diagnosed, did you use any of the following substances without a prescription from a healthcare professional? (Options include illicit drugs) Before being diagnosed with SJS or TEN, did you eat anything that was not usually in your normal diet? If so what was it, and did your doctor believe that this food item might have contributed to you getting SJS or TEN?

* The same questions were asked for parents taking the survey for a child who was diagnosed with SJS, SJS-TEN, or TEN.

**Table 2:** Summary of respondent demographics.

Characteristic	Number of Respondents
**Age, years**	**(*n* = 251)**
Mean (range)	43 (4–87)
Age Category (*n*, %)	
0–10	86 (34.3)
11–20	18 (7.2)
21–30	23 (9.2)
31–40	26 (10.4)
41–50	27 (10.8)
51–60	49 (19.5)
61–70	19 (7.6)
71–80	2 (0.8)
81–90	1 (0.4)
**Gender (*n*, %)**	**(*n* = 173)**
Female	121 (70)
Male	52 (30)
**Race (*n*, %)**	**(*n* = 135)**
White	115 (85.2)
Black or African American	9 (6.67)
Asian	5 (3.7)
American Indian or Alaska Native	2 (1.5)
Other	7 (5.2)
Unknown	1 (0.74)
**Ethnicity (*n*, %)**	**(*n* = 169)**
Hispanic	13 (7.7)
**Comorbidities (*n*, %)**	**(*n* = 66)**
Herpes	11 (16.7)
Pneumonia	11 (16.7)
HIV	2 (3)
AIDS	1 (1.5)
Hepatitis	2 (3)
Erythema multiforme	13 (19.7)
MIRM	7 (10.6)
Other Comorbidities	36 (54.5)

HIV: human immunodeficiency virus; AIDS: acquired immunodeficiency syndrome; MIRM: mycoplasma pneumonia-induced rash and mucositis.
